# Collapsin Response Mediator Protein 2 (CRMP2) Modulates Induction of the Mitochondrial Permeability Transition Pore in a Knock-In Mouse Model of Alzheimer’s Disease

**DOI:** 10.3390/cells15020179

**Published:** 2026-01-19

**Authors:** Tatiana Brustovetsky, Rajesh Khanna, Nickolay Brustovetsky

**Affiliations:** 1 Department of Biochemistry, Molecular Biology, and Pharmacology, School of Medicine, Indiana University, Indianapolis, IN 46202, USA; 2 Center for Advanced Pain Therapeutics and Research (CAPToR), College of Medicine University of Florida, Gainesville, FL 32610, USA; r.khanna@ufl.edu; 3 Department of Pharmacology & Therapeutics, and McKnight Brain Institute, College of Medicine University of Florida, Gainesville, FL 32610, USA; 4 Stark Neurosciences Research Institute, Indiana University School of Medicine, Indianapolis, IN 46202, USA

**Keywords:** CRMP2, Alzheimer’s disease, mitochondria, adenine nucleotide translocase, permeability transition pore, (S)-lacosamide, cortical neurons

## Abstract

Hyperphosphorylated collapsin response mediator protein 2 (CRMP2) is elevated in the cerebral cortex of an APP-SAA knock-in mouse model of Alzheimer’s disease and binds the adenine nucleotide translocase (ANT) in a phosphorylation-dependent manner. We propose that, in Alzheimer’s disease (AD) mitochondria, dissociation of hyperphosphorylated CRMP2 from ANT promotes opening of the permeability transition pore (PTP). We showed that purified ANT, when reconstituted into giant liposomes, forms large calcium-dependent channels resembling the PTP, which are effectively blocked by recombinant, unphosphorylated CRMP2. In synaptic mitochondria isolated from the cortices of APP-SAA knock-in mice and control B6J hAbeta mice, we observed an increased susceptibility to permeability transition pore (PTP) induction in AD mitochondria, accompanied by reduced viability of cultured cortical neurons. Pre-treatment of AD mice with the CRMP2-binding small molecule (S)-lacosamide ((S)-LCM), which prevents CRMP2 hyperphosphorylation and restores its interaction with ANT, attenuated PTP induction and improved neuronal viability. Interestingly, direct application of (S)-LCM to isolated mitochondria failed to suppress PTP induction, indicating that its protective effect requires upstream cellular mechanisms. These findings support a phosphorylation-dependent role for CRMP2 in regulating PTP induction in AD mitochondria and highlight (S)-LCM as a promising therapeutic candidate for mitigating mitochondrial dysfunction and enhancing neuronal viability in AD.

## 1. Introduction

Alzheimer’s disease (AD) is a severe neurodegenerative disorder with no effective cure. Despite decades of intensive research, its underlying mechanisms remain incompletely understood, in part due to the complexity of its pathological processes. While early breakthroughs—such as the identification of amyloid-β (Aβ) and tau—shaped the field over 25 years ago, progress since then has been incremental, underscoring the urgent need for novel insights into AD pathogenesis and therapeutic intervention [1].

The search for molecular drivers of AD has long centered on hallmark pathologies such as amyloid-β plaques and tau tangles. Yet, emerging evidence suggests that disruptions in neuronal signaling and cytoskeletal dynamics may play a pivotal role in disease onset and progression. Among the proteins implicated in these processes is Collapsin Response Mediator Protein 2 (CRMP2), a cytosolic phosphoprotein originally characterized for its role in axonal guidance and neurite outgrowth [2]. Increasing evidence implicates CRMP2 in the pathogenesis of AD [3,4,5,6], where dysregulated phosphorylation mediated by hyperactive kinases such as glycogen synthase kinase-3β (GSK-3β) and cyclin-dependent kinase 5 (Cdk5) contributes to disease-associated cellular abnormalities [7,8,9,10]. Notably, phosphorylation of CRMP2 at specific residues targeted by these kinases is markedly elevated in postmortem AD brains [11,12,13,14,15] and in transgenic mouse models of the disease [13,15,16]. Intriguingly, this hyperphosphorylation is detectable as early as two months of age in AD mice, preceding overt neuropathological symptoms and suggesting a potential initiating role in disease development [13]. Despite these associations, the functional consequences of CRMP2 hyperphosphorylation remain poorly understood. How this post-translational modification contributes to mitochondrial dysfunction and neuronal vulnerability in AD is an open question—one that may hold the key to novel therapeutic strategies.

Although traditionally viewed as a cytosolic protein, CRMP2 has recently been found to associate with mitochondria [17,18], with a subset localized to the intermembrane space between the inner and outer mitochondrial membranes [18]. Within this compartment, CRMP2 interacts with the adenine nucleotide translocase 1 (ANT) [17], a key regulator of mitochondrial bioenergetics [19]. Notably, this interaction is disrupted by phosphorylation of CRMP2—a modification mediated by kinases such as GSK-3β and Cdk5, both of which are hyperactive in AD. Elevated CRMP2 phosphorylation, observed in both human AD brains and transgenic mouse models [11,12,13,14,15,16], suggests that CRMP2 dissociation from the ANT may be an early event in AD pathogenesis. This may lead to reduced ANT activity [20]. However, other potential consequences of this dissociation remain unclear. It remains unclear whether preventing CRMP2 hyperphosphorylation and maintaining its association with the ANT can safeguard mitochondrial integrity and support neuronal survival in AD.

Mitochondria play a central role in buffering cytosolic calcium [21], especially during periods of neuronal activity. However, excessive mitochondrial calcium uptake can trigger an induction of the mitochondrial permeability transition pore (PTP)—a catastrophic event that leads to membrane depolarization, organelle swelling, impaired ATP synthesis, rupture of the outer mitochondrial membrane, and release of pro-apoptotic factors [21,22,23]. Notably, the propensity for PTP induction is elevated in Alzheimer’s disease (AD), potentially driven by interactions between amyloid-β (Aβ) and mitochondrial components such as cyclophilin D and the oligomycin sensitivity conferring protein (OSCP) of the F_1_F_0_-ATP synthase complex [24,25,26,27,28,29,30]. While these mechanisms have been implicated in AD-related mitochondrial dysfunction, the precise molecular events that facilitate PTP induction remain incompletely understood. This uncertainty leaves open the possibility that additional, yet unidentified, pathways contribute to mitochondrial vulnerability in AD.

In prior work, we identified bioenergetic deficits in cortical synaptic mitochondria from APP-SAA knock-in mice, a genetically faithful model of AD, which were linked to hyperphosphorylation of CRMP2 and its dissociation from the ANT [20], a key component of the mitochondrial PTP [31,32,33,34,35]. Pharmacological intervention with the small molecule (S)-LCM prevented CRMP2 hyperphosphorylation, preserved its interaction with the ANT, and restored mitochondrial oxidative metabolism [20]. Given the central role of the ANT in PTP regulation [31,32,33,34,35], these findings raised the possibility that CRMP2 phosphorylation status may influence PTP susceptibility in AD mitochondria. In this study, we directly tested this hypothesis and uncovered a previously unrecognized role for CRMP2 in modulating PTP induction, revealing a potential therapeutic axis for mitochondrial protection in AD.

## 2. Materials and Methods

### 2.1. Animals

All experimental procedures involving animals were conducted in accordance with the U.S. National Institutes of Health guidelines for the care and use of laboratory animals and were approved by the Institutional Animal Care and Use Committee at Indiana University School of Medicine (protocol #23156 MD/R/E). We employed APP-SAA knock-in (KI) (AD mice) mice [36] (Jackson Laboratory, Bar Harbor, ME, USA, strain #034711), which harbor a humanized Aβ sequence incorporating multiple familial Alzheimer’s disease mutations: R684H, F681Y, G676R in the Aβ region, KM670/671NL (Swedish) in exon 16, and E693G (Arctic) and T714I (Austrian) in exon 17 of the endogenous mouse App gene. These mice model AD pathology without the confounding effects of APP overexpression. Age-matched B6J hAbeta mice (Jackson Laboratory, strain #033013), which express a humanized Aβ1–42 region but lack pathogenic mutations, served as controls. Both male and female mice were included in all experimental groups. Breeding and housing were maintained at the Laboratory Animal Resource Center, Indiana University School of Medicine. The APP-SAA KI model was selected for its physiological relevance and reduced susceptibility to artifacts commonly observed in APP-overexpressing transgenic lines [36].

### 2.2. Mouse Oral Gavage

To administer compounds, mice received daily oral gavage for 7 consecutive days. (S)-LCM was delivered at a dose of 10 mg/kg body weight, dissolved in dimethyl sulfoxide (DMSO) and diluted in 0.2 mL sterile saline. Control animals received vehicle alone (10 μL DMSO in 0.2 mL saline). Gavage was performed using a precision 0.5 mL syringe fitted with a 20-gauge, ball-tipped feeding needle designed for murine oral delivery (FN-7910, Roboz Surgical Instrument Co., Gaithersburg, MD, USA). All procedures were conducted under standardized conditions to minimize stress and ensure consistent dosing.

### 2.3. Isolation and Purification of Brain Synaptic Mitochondria

Cortical synaptic mitochondria were isolated using a previously established protocol involving a discontinuous Percoll gradient (24%/40%), as described in detail elsewhere [18]. This method yields highly purified mitochondrial fractions enriched for synaptic populations. The integrity and purity of the resulting preparations have been validated in our prior work [18], confirming minimal contamination from non-mitochondrial compartments and suitability for downstream biochemical analyses.

### 2.4. Mitochondrial Swelling and Membrane Potential

Mitochondrial swelling was quantified by tracking changes in light scattering at 525 nm within a thermostated (37 °C), continuously stirred 0.3 mL optical chamber. The detection geometry was configured in a backscatter arrangement, with the photodetector positioned directly opposite the incident beam (180°), enhancing sensitivity to volumetric changes in the mitochondrial suspension. Experiments were conducted in a defined KCl-based buffer composed of 125 mM KCl, 0.5 mM MgCl_2_, 3 mM KH_2_PO_4_, 10 mM HEPES (pH 7.4), 10 μM EGTA, 3 mM pyruvate, and 1 mM malate. This medium supported simultaneous monitoring of mitochondrial swelling and membrane potential. Swelling was inferred from a reduction in light scattering, reflecting increased mitochondrial volume. Alamethicin (30 μg/mL), a pore-forming peptide [37], was used to induce maximal swelling, which served as the reference (100%) for normalization [38]. Calcium-induced swelling responses were expressed as a percentage of this alamethicin-defined maximum. To evaluate mitochondrial membrane potential, we employed a tetraphenylphosphonium (TPP^+^)-selective electrode, enabling real-time quantification of TPP^+^ distribution between the incubation medium and the mitochondrial matrix [39]. A rise in TPP^+^ concentration in the medium indicated membrane depolarization, whereas a decline signified polarization [38].

### 2.5. Mitochondrial Ca^2+^ Retention Capacity

Mitochondrial Ca^2+^ uptake was monitored in a 0.3 mL thermostated chamber maintained at 37 °C under continuous stirring, using a miniature Ca^2+^-selective electrode as previously described [38]. The assay was performed in a KCl-based buffer supplemented with 3 mM pyruvate, 1 mM malate, 0.1 mM ADP, and 1 μM oligomycin to support oxidative metabolism while inhibiting ATP synthase. In experiments with bongkrekic acid (BKA), ADP (and oligomycin) were omitted to avoid interference with the ANT. Calcium was introduced into the medium as sequential 10 μM CaCl_2_ pulses. Uptake was inferred from the progressive decline in free Ca^2+^ concentration in the incubation medium, reflecting mitochondrial sequestration. Quantification was expressed as calcium retention capacity, normalized to mitochondrial protein content (μmol Ca^2+^/mg protein) [38].

### 2.6. Immunoblotting

Brain tissues were lysed on ice using a buffer composed of 50 mM Tris-HCl (pH 7.4), 150 mM NaCl, 1% NP-40, 0.1% SDS, and 1 mM EDTA. Protease and phosphatase activities were inhibited by including commercial inhibitor cocktails (Roche, Indianapolis, IN, USA; Cat. #04906845001 and #04693124001). Homogenates were incubated on ice for 30 min and subsequently centrifuged at 100,000× *g* for 30 min at 4 °C. The resulting supernatant was collected for SDS-PAGE analysis. Protein samples (20 µg per lane) were resolved on 4–12% Bis-Tris polyacrylamide gels (Invitrogen (Carlsbad, CA, USA), Cat. #NP0335) and transferred to Hybond-ECL nitrocellulose membranes (Amersham Biosciences, (Piscataway, NJ, USA), Cat. #RPN78D). Membranes were blocked for 1 h at room temperature in phosphate-buffered saline (PBS, pH 7.2) containing 5% non-fat dry milk and 0.15% Triton X-100. Primary antibody incubations were performed overnight at 4 °C using rabbit anti-CRMP2 (Sigma, Cat. #C2993, 1:1000) or rabbit anti-ANT 1/2 (Proteintech, (Rosemont, IL, USA), Cat. #15997-1, 1:1000). After blocking, membranes were incubated with the following primary antibodies: sheep anti-CRMP2 pThr509/514 (Kinasource, Dundee, Scotland, UK; Cat. #PB-043; 1:1500), rabbit anti-CRMP2 pSer522 (ECM Biosciences, Versailles, KY, USA; Cat. #CP2191; 1:1500), rabbit anti-CRMP2 (Sigma-Aldrich, St. Louis, MO, USA; Cat. #C2993; 1:1000), rabbit anti-ANT1/2 (Proteintech, Rosemont, IL, USA; Cat. #15997-1; 1:1000), and mouse anti-GAPDH (Abcam, Cambridge, MA, USA; Cat. #ab9484; 1:2000). Blots were then incubated with horseradish peroxidase-conjugated secondary antibodies (goat anti-mouse or goat anti-rabbit IgG; Jackson ImmunoResearch Laboratories, West Grove, PA, USA, 1:25,000 or 1:20,000, respectively) for 1 h at room temperature. Signal detection was carried out using Supersignal West Pico chemiluminescent substrate (Pierce, (Rockford, IL, USA), Cat. #32106). Molecular weight markers were visualized using PageRuler Plus Prestained Protein Ladder (Thermo Fisher, (Waltham, MA, USA), Cat. #26619; 5 µL per lane). Immunoblot images were inverted, and band intensities were quantified by integrated density analysis following background subtraction using Adobe Photoshop (v22.2.0).

### 2.7. Co-Immunoprecipitation

Synaptic mitochondria were isolated from the cerebral cortex of 4-month-old APP-SAA knock-in mice using Percoll gradient fractionation. Animals were administered either (S)-LCM (10 mg/kg body weight, oral gavage for 7 days), vehicle (10 µL DMSO in 0.2 mL saline, oral gavage for 7 days), or received no treatment. Age-matched B6J hAβ mice were used as controls. Isolated mitochondria were lysed in buffer containing 125 mM KCl, 3 mM KH_2_PO_4_, 0.5 mM MgCl_2_, 10 mM HEPES (pH 7.4), 1% NP-40, 0.1% SDS, and protease inhibitors (Roche). Lysates were pre-cleared with Protein A/G agarose beads (Santa Cruz Biotechnology, Dallas, TX, USA, Cat # sc-2002) for 2 h at 4 °C, then incubated overnight at 4 °C with rabbit anti-CRMP2 (Sigma, Cat # C2993; 1:1000) or rabbit anti-ANT1/2 (Proteintech, Cat # 15997-1; 1:1000) under gentle agitation. Immunocomplexes were captured with Protein A/G beads for 2 h at 4 °C, washed three times with lysis buffer, and eluted by heating at 70 °C in SDS loading dye (Invitrogen). Proteins (20 µg per lane) were resolved on 3–8% Tris–acetate gels (Invitrogen, Cat # EA0375BOX) and immunoblotted as described previously [40]. Membranes were probed with anti-CRMP2 or anti-ANT1/2 (1:1000) and reprobed for bait protein controls to confirm immunoprecipitation efficiency. Input loading controls included anti-VDAC1 (Calbiochem; 1:1000) for CRMP2 and anti-Complex II 70 kDa subunit (Invitrogen; 1:1000) for ANT1/2. Immunoblot images were inverted, and band intensities quantified after background subtraction using Adobe Photoshop 22.2.0. All blots represent at least three independent experiments.

### 2.8. Mitochondrial Isolation and ANT Reconstitution in Proteoliposomes

Mitochondria were extracted from the brains of ten adult C57BL/6J mice using protocols previously established in our laboratory [18]. To purify the adenine nucleotide translocase (ANT), we adapted methodologies originally developed for yeast mitochondria [41], applying them to mouse brain-derived mitochondrial preparations. For reconstitution of the mouse brain ANT into proteoliposomes, we followed a strategy previously validated for yeast ANT [41]. A lipid mixture was prepared by dissolving 40 mg of phosphatidylcholine (Millipore-Sigma, (Burlington, MA, USA), Cat # 3356) and 1.5 mg of cardiolipin (Millipore-Sigma, Cat # C0563) in 0.45 mL of a detergent-rich buffer containing 11% (*w*/*v*) C_12_E_8_ (Millipore-Sigma, Cat # P8925), 87 mM Na_2_SO_4_, 1 mM EGTA, and 175 mM Tricine-OH (pH 8.0). This lipid solution was then combined with 1.5 mL of the ANT extract, yielding a final protein-to-phospholipid weight ratio of 0.015. The resulting mixture contained 2.5% (*w*/*v*) C_12_E_8_, 20 mM Na_2_SO_4_, 0.23 mM EGTA, 40.4 mM Tricine-OH (pH 8.0), and 9.6 mg/mL phospholipid. Proteoliposome formation was initiated by gradual detergent removal using Amberlite XAD-4 ion exchange beads (Millipore-Sigma, Cat # XAD4). To eliminate residual external components, the proteoliposomes were subsequently passed through a Sephadex G-75 column (30 × 1 cm; Millipore-Sigma, Cat # G75120) pre-equilibrated with a buffer containing 100 mM sucrose, 30 mM Na_2_SO_4_, 1 mM Tricine-OH (pH 7.5), and 1 mM EDTA.

### 2.9. Giant Liposomes

Giant liposomes were generated using a modified dehydration–rehydration technique based on protocols originally developed by Criado and Keller [42] and Schwarz et al. [43], with further refinements as described in our previous studies [32,33]. To ensure sterility and prevent microbial contamination, all solutions used during ANT reconstitution and liposome preparation were filtered through 0.2 μm Millipore membranes. Small ANT-containing proteoliposomes were diluted tenfold with azolectin vesicles, which were prepared from acetone-washed azolectin (type S-II, Sigma). Azolectin (2 mg) was first dissolved in 1 mL of chloroform, evaporated under a nitrogen stream, and rehydrated in 350 μL of buffer containing 100 mM KCl and 5 mM Hepes (pH 7.2). The resulting lipid suspension was extruded sequentially through polycarbonate membranes with pore sizes of 800 nm, 400 nm, and 200 nm using a LiposoFast™ extruder (Avestin Inc., Ottawa, ON, Canada), following established procedures [44]. To form giant liposomes, 5 μL of the vesicle suspension were placed on a glass slide and dehydrated under vacuum in a desiccator containing anhydrous CaCl_2_ for 5 min at room temperature. Rehydration was performed overnight at 4 °C using 10 μL of a buffer composed of 50 mM KCl and 2.5 mM Hepes (pH 7.2). This process yielded giant liposomes with diameters ranging from 20 to 60 μm, suitable for patch-clamp electrophysiological recordings.

### 2.10. Patch Clamping

Patch-clamp recordings in the excised patch configuration were conducted following previously established protocols [32,33]. Micropipettes were fabricated from 1.5 mm outer diameter borosilicate glass capillaries with an internal filament (Sutter Instruments (Novato, CA, USA) Cat # BF150-86-10) using a P-1000 micropipette puller (Sutter Instruments). The pipettes were filled with a standard bath solution composed of 100 mM KCl, 2 mM MgCl_2_, 0.5 mM CaCl_2_, 4 mM potassium gluconate, 5 mM MES, and 5 mM Tris, adjusted to pH 7.4. The resulting pipette resistance ranged between 3–5 MΩ. For patch formation, 5 μL of giant liposomes containing the ANT were introduced into a 3.0 mL recording chamber pre-filled with the same standard bath solution. Liposomes adhered to the chamber base were gently contacted with the pipette tip to initiate seal formation. A high-resistance seal was typically achieved either spontaneously upon contact or with minimal negative pressure applied via the pipette. Recordings were performed at ambient temperature (~22 °C) using a HEKA EPC-10 amplifier system. Data acquisition and analysis were carried out using PatchMaster v2x80 software (HEKA Elektronik, Holliston, MA, USA). Currents were low-pass filtered at 3.3 kHz and digitized at a sampling rate of 5 kHz. All voltages are reported relative to the pipette electrode potential.

### 2.11. Recombinant CRMP2 Protein Expression and Purification

Recombinant CRMP2 protein was produced using established bacterial expression protocols [45]. Sequence-verified pGex-Glu-CaV2.2-type channel constructs were introduced into *Escherichia coli* BL21 (DE3) cells for protein expression. The CRMP2-GST fusion plasmids were generously provided by Dr. Akihiro Kurimasa (Tottori, Japan). Protein induction was initiated with 1 mM isopropyl-β-D-thiogalactopyranoside (IPTG). Following induction, cultures were grown overnight at 16 °C and processed to yield CRMP2-enriched lysates prepared in Tris-based buffer containing 20 mM Tris (pH 7.5), 200 mM NaCl, 0.1 mM EDTA, 1 mM dithiothreitol (DTT), and a protease inhibitor cocktail. Cells were disrupted using an M-110L microfluidizer (Microfluidics Corp., Newton, MA, USA), and detergent extraction with 1% Triton X-100 followed by high-speed centrifugation removed insoluble material. The resulting supernatant was concentrated to 30 mg/mL, and CRMP2-containing preparations were flash-frozen and stored at −80 °C in 25 mM Tris-HCl, 100 mM glycine (pH 7.3), and 10% glycerol for subsequent biochemical analyses [46].

### 2.12. Statistics

Quantitative data are reported as the mean ± standard deviation (SD), with the number of independent experiments specified in the figure legends. Statistical comparisons were performed using either unpaired two-tailed *t*-tests or one-way analysis of variance (ANOVA), followed by Bonferroni’s multiple comparison post hoc test where appropriate. All analyses were conducted using GraphPad Prism^®^ software (version 4.0; GraphPad Software Inc., La Jolla, CA, USA). Each dataset was derived from distinct preparations of isolated mitochondria or independently cultured neuronal samples.

## 3. Results

**Figure 1:**
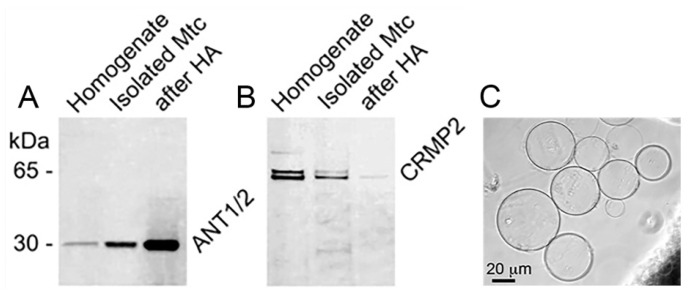
ANT purification with hydroxyapatite chromatography (**A**) and removal of CRMP2 (**B**). Giant liposomes used in our patch-clamp experiments (**C**). In (**A**,**B**), typical immunoblots demonstrating ANT enrichment in the brain tissue lysates from B6J hAbeta mice following purification with hydroxyapatite (HA) chromatography ((**A**), after HA) and removal of CRMP2 from this solution ((**B**), after HA). Mtc, mitochondria. In (**C**), the giant liposomes with reconstituted ANT were prepared as we described previously [32,33] and measured approximately 40–60 μm in diameter.

The adenine nucleotide translocase (ANT), a central component of the mitochondrial permeability transition pore (PTP) [31,32,33,34,35], is known to form large, Ca^2+^-activated ion channels in phospholipid membranes [32,33]. Given prior evidence of CRMP2 interaction with the ANT [20], we hypothesized that CRMP2 may modulate ANT channel activity and thereby influence the likelihood of PTP induction. To test this, we reconstituted purified ANT into giant liposomes. The ANT was isolated from brain lysates using hydroxyapatite chromatography, which yielded a strong enrichment of ANT protein (Figure 1A) while depleting CRMP2 below detectable levels (Figure 1B). This allowed us to assess ANT channel activity in a CRMP2-free environment. In our experiments, we generated and used giant ANT-reconstituted liposomes with diameters of 20–60 μm, which were suitable for electrophysiological patch-clamp studies (Figure 1C).

**Figure 2:**
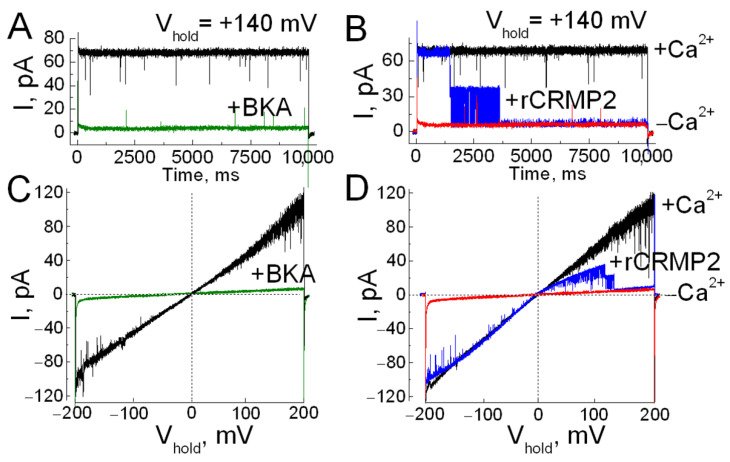
Electrophysiological characterization of ANT channel activity and modulation by recombinant CRMP2. (**A,B**) Representative ion current traces recorded from excised patches of giant liposomes reconstituted with ANT, under a step voltage protocol at a holding potential (V_hold_) of +140 mV. Currents were recorded in the presence of 0.5 mM CaCl_2_ (black traces), with 1 µM bongkrekic acid (Millipore-Sigma, Cat # B6179) (BKA; green traces), with 10 µg/mL recombinant CRMP2 (rCRMP2; blue traces), added to both bath and pipette solutions, and in the absence of Ca^2+^ in the bath solution (red traces). (**C**,**D**) Representative ion currents recorded using a voltage ramp protocol ranging from −200 mV to +200 mV over 10 s, under the same experimental conditions as in panels (**A**,**B**). These recordings illustrate the modulatory effects of rCRMP2 and BKA on ANT channel activity and the calcium dependence of the observed currents.

Patch-clamp recordings from ANT-containing liposomes revealed a prominent Ca^2+^-induced ion channel (Figure 2), consistent with previously described Ca^2+^-dependent ANT pores [32,33]. Channel activity was abolished by bongkrekic acid (BKA), a selective ANT inhibitor and known PTP suppressor [19,32,33,47]. The ANT-related ion currents revealed voltage gating behavior and underwent a closure at high positive voltage (Figure 2D). Notably, application of recombinant unphosphorylated CRMP2 (rCRMP2) led to rapid channel inactivation (Figure 2B,D), suggesting that CRMP2 directly interacts with the ANT pore to block ion conductance. These findings support a model in which CRMP2 binding to ANT suppresses pore formation and may thereby reduce the likelihood of PTP induction.

**Figure 3:**
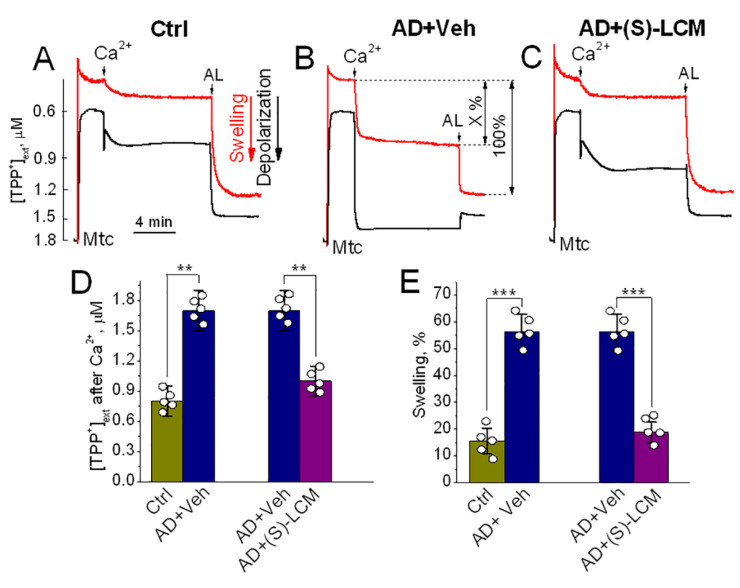
Induction of the PTP in synaptic mitochondria isolated from cortices of 4-month old APP-SAA KI (AD) and B6J hAbeta (Control, Ctrl) mice. Mitochondria were isolated and PTP induction was assessed as we described previously [38]. Here and in Figure 4, mitochondria were incubated at 37 °C in KCl-based medium supplemented with 1 mM malate plus 3 mM pyruvate [38]. In (**A**), mitochondria from Ctrl mice. AL, alamethicin a pore-forming agent [37], causing maximal (100%) swelling [38]. In (**B**) and (**C**), AD mice were pretreated with either a vehicle (Veh, 10 µL of DMSO in 0.2 mL saline) or 10 mg (S)-LCM/kg, delivered by oral gavage for 7 days prior to experiment, respectively. In (**D**) and (**E**), statistical summary of changes in external TPP^+^ concentration ([TPP^+^]_ext_) and light scattering, respectively. Where indicated, 50 μM Ca^2+^ was applied. Mitochondrial swelling was monitored by following light scattering of mitochondrial suspension (red traces) at 525 nm; mitochondrial membrane potential was followed with a tetraphenylphosphonium (TPP^+^)-sensitive electrode (black traces) [38]. A decrease in light scattering is indicative of mitochondrial swelling, whereas an increase in [TPP^+^]_ext_ is indicative of mitochondrial depolarization. Mitochondria were isolated from 4-month old AD and Ctrl mice of both sexes. Data are mean ± SD, N = 5 separate experiments, ** *p* < 0.01, *** *p* < 0.001.

To evaluate this mechanism in a physiological context, we examined synaptic mitochondria isolated from APP-SAA KI mice (AD) and B6J hAbeta mice (Control, Ctrl). Upon Ca^2+^ challenge, AD mitochondria exhibited significantly greater depolarization and swelling, as measured by changes in membrane potential and light scattering at 525 nm [38] (Figure 3A,B,D). These responses are indicative of increased proclivity to PTP induction.

We next tested whether pharmacological modulation of CRMP2 phosphorylation could mitigate this vulnerability. Oral administration of (S)-LCM (10 mg/kg for 7 days) reduced CRMP2 phosphorylation, preserved CRMP2–ANT interaction (Supplementary Figures S1 and S2) [20], and protected AD mitochondria from Ca^2+^-induced PTP activation (Figure 3C,D). In contrast, direct application of (S)-LCM to isolated mitochondria failed to prevent PTP induction (Supplementary Figure S3), suggesting that its protective effect requires intact cellular signaling or upstream modulation of CRMP2 phosphorylation.

**Figure 4:**
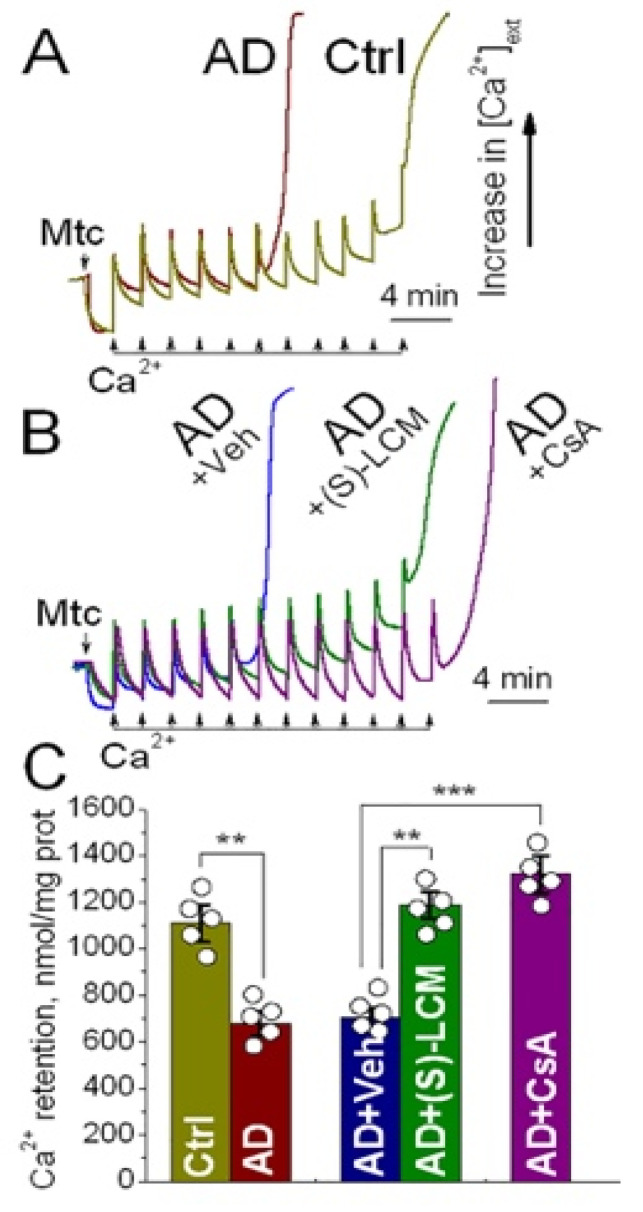
Ca^2+^ retention capacity of synaptic mitochondria isolated from cortices of 4-month old APP-SAA KI (AD) and B6J hAbeta (Control, Ctrl) mice. The Ca^2+^ retention capacity was evaluated as we described previously in the KCl-based incubation medium supplemented with 1 mM malate plus 3 mM pyruvate [38]. In (**A**), synaptic mitochondria from AD and Ctrl mice. In (**B**), where indicated, mitochondria were isolated from AD mice pretreated with either a vehicle (Veh, 10 µL of DMSO in 0.2 mL saline) or 10 mg/kg (S)-LCM delivered by oral gavage for 7 days prior to mitochondrial isolation, or 1 μM cyclosporin A (CsA, positive control) was added to isolated mitochondria during the experiment. Ca^2+^ uptake and Ca^2+^ retention by mitochondria were monitored by following Ca^2+^ concentration in the medium ([Ca^2+^]_ext_) with a Ca^2+^-selective electrode [38]. To assess mitochondrial Ca^2+^ retention capacity, multiple pulses of 10 µM Ca^2+^ were applied to mitochondria until a failure to take Ca^2+^ up and the release of accumulated Ca^2+^. In (**C**), statistical summary. Data are mean ± SD, ** *p* < 0.01, *** *p* < 0.001, N = 5 separate experiments.

To further substantiate these findings, we measured mitochondrial Ca^2+^ retention capacity—a functional readout inversely correlated with PTP susceptibility [48]. AD mitochondria retained significantly less Ca^2+^ than Controls (Figure 4A,C), consistent with their heightened propensity to PTP induction. (S)-LCM treatment of AD mice restored Ca^2+^ retention capacity in AD mitochondria (Figure 4B,C), while (S)-LCM treatment of Control mice had negligible effects on mitochondrial Ca^2+^ retention capacity (Supplementary Figure S4). Direct application of (S)-LCM to isolated AD mitochondria had no effect (Supplementary Figure S5), again pointing to an indirect mechanism of action. As a positive control, cyclosporin A (CsA), a well-established PTP inhibitor [49,50], significantly increased Ca^2+^ retention capacity in AD (Figure 4B,C) and Control mitochondria (Supplementary Figure S4). Importantly, CsA failed to further enhance Ca^2+^ retention in mitochondria from (S)-LCM-treated AD mice (Figure 4B,C), suggesting that both interventions converge on the same pathway to suppress PTP induction.

**Figure 5:**
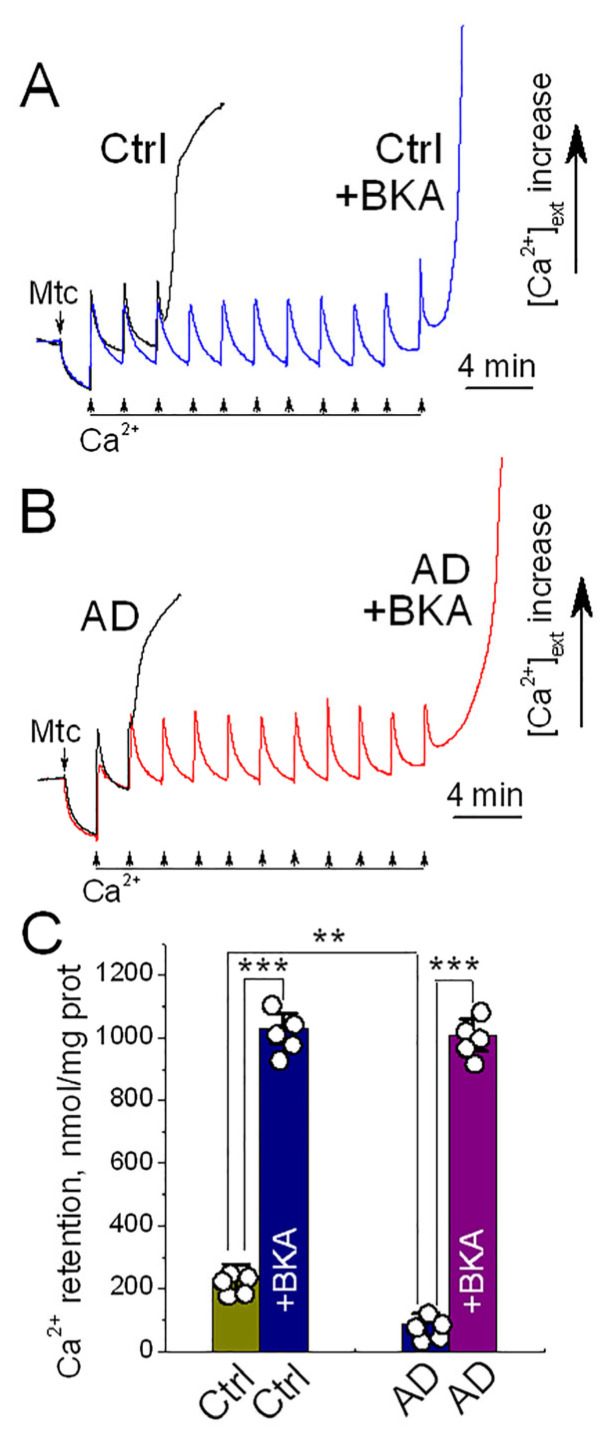
Ca^2+^ retention capacity of synaptic mitochondria from cortical tissue of 4-month-old APP-SAA KI (AD) and B6J hAβ (Control) mice: the effect of bongkrekic acid (BKA). Synaptic mitochondria were incubated in a KCl-based medium supplemented with 1 mM malate and 3 mM pyruvate, without ADP or oligomycin, as previously described [38]. Ca^2+^ uptake and retention were monitored by measuring extracellular Ca^2+^ concentration ([Ca^2+^]_ext_) using a Ca^2+^-selective electrode [38]. To determine retention capacity, sequential 10 μM Ca^2+^ pulses were applied until mitochondria failed to take up additional Ca^2+^ and released accumulated Ca^2+^. Panels (**A**) and (**B**) show representative traces from synaptic mitochondria of control and AD mice, respectively. Where indicated, mitochondria were treated with 5 μM BKA. Panel (**C**) summarizes Ca^2+^ retention capacity across groups. Data are presented as mean ± SD; ** *p* < 0.01, *** *p* < 0.001; N = 5 independent experiments.

To determine whether the Ca^2+^-induced PTP in Control and AD mitochondria was associated with the ANT, we employed bongkrekic acid (BKA), a specific ANT inhibitor and negative modulator of the PTP [19,32,33,47]. ADP and oligomycin were omitted from these experiments to prevent interference with the ANT. Under these conditions, mitochondria accumulated substantially less Ca^2+^ compared to those incubated with ADP and oligomycin (compare Figure 4 and Figure 5). Nevertheless, AD mitochondria retained only about half as much Ca^2+^ as control mitochondria (Figure 5). Treatment with BKA markedly increased and equalized Ca^2+^ retention capacity between the two groups. These results suggest that the Ca^2+^-induced PTP observed in our experiments is closely linked to the ANT.

**Figure 6:**
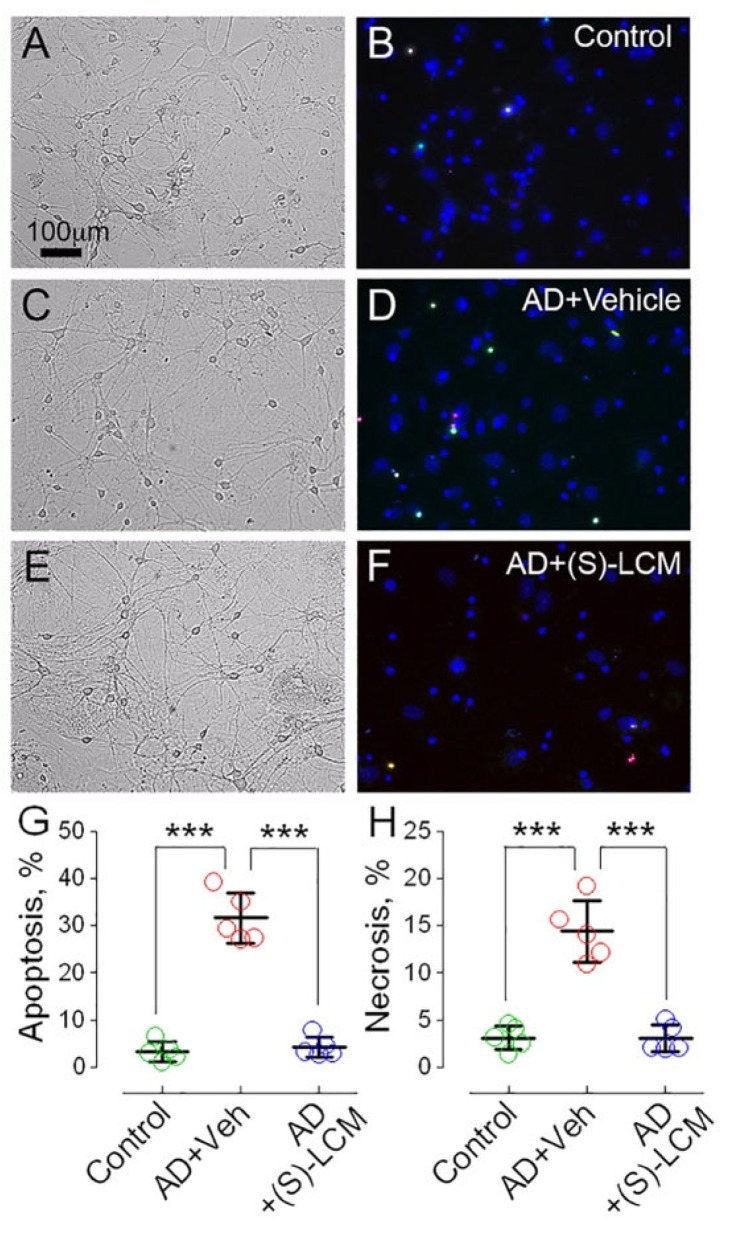
Differential apoptotic and necrotic cell death in cortical neurons from APP-SAA KI (AD model) and B6J hAbeta (Control) mice. Primary cortical neurons were cultured for 21 days and stained with Hoechst 33342 (blue, nuclear marker), YO-PRO-1 (green, apoptotic marker), and propidium iodide (red, necrotic marker) to assess cell death phenotypes. (**A**,**B**), representative bright field and fluorescence micrographs of neurons derived from Control mice. (**C**,**D**), neurons from APP-SAA KI mice treated with vehicle (Veh; 0.01% DMSO) for 7 days prior to analysis show increased apoptotic and necrotic markers. (**E**,**F**), neurons from APP-SAA KI mice incubated with 10 µM (S)-LCM for 7 days exhibit reduced cell death, indicating neuroprotective effect. (**G**,**H**), quantitative analysis of apoptotic and necrotic cell populations across experimental conditions. Data represent mean ± SD from five independent experiments using neurons from separate platings. Statistical significance was determined by one-way ANOVA followed by post hoc testing; *** *p* < 0.001.

Given the link between PTP induction and cell viability, we next asked whether increased PTP sensitivity in AD mitochondria correlates with neuronal vulnerability. In previous work, we showed that cortical neurons from double-transgenic APP/PS1 mice were more prone to cell death than wild-type neurons [51]. Extending this observation, we found that cultured cortical neurons from APP-SAA KI mice exhibited significantly higher cell death compared to neurons from B6J hAbeta controls (Figure 6). While these findings may not directly reflect in vivo neurodegeneration, they suggest that AD neurons are under greater stress in vitro. Notably, treatment with (S)-LCM (10 μM in culture medium for 7 days) conferred neuroprotection, significantly reducing cell death in AD neurons (Figure 6).

Together, our results support a model in which CRMP2 regulates ANT channel activity and PTP induction. Pharmacological preservation of CRMP2–ANT interaction via (S)-LCM confers mitochondrial and neuronal resilience in AD models, highlighting a potential therapeutic strategy for mitigating mitochondrial dysfunction and neurodegeneration.

## 4. Discussion

In our previous work, we identified CRMP2 hyperphosphorylation in APP-SAA knock-in (KI) mice, which coincided with its dissociation from the adenine nucleotide translocase (ANT) and a reduction in ANT-mediated ADP/ATP transport activity [20]. Beyond its canonical role in ADP/ATP exchange across the inner mitochondrial membrane, the ANT is also implicated in the formation of the mitochondrial permeability transition pore (PTP) [23,31,32,33,35,47,52,53,54,55,56,57,58], a pathological event that compromises mitochondrial integrity [59]. These findings led us to hypothesize that CRMP2 dissociation from the ANT may sensitize mitochondria to PTP induction in Alzheimer’s disease (AD). In the present study, we provide direct evidence that CRMP2 modulates PTP susceptibility in a phosphorylation-dependent manner. Specifically, cortical synaptic mitochondria from APP-SAA KI mice exhibited an increased propensity for PTP induction, which correlated with CRMP2 hyperphosphorylation and its disengagement from the ANT. Notably, pharmacological inhibition of CRMP2 hyperphosphorylation using (S)-LCM [20] mitigated PTP induction, revealing a previously unrecognized mechanism of PTP regulation in AD mitochondria.

PTP induction is known to be elevated in AD [24,25,26], potentially driven by interactions between amyloid-β (Aβ) and mitochondrial proteins such as cyclophilin D or the oligomycin sensitivity conferring protein (OSCP) of the F_1_F_0_-ATP synthase complex [24,25,26,27,28,29,30]. Activation of the PTP results in mitochondrial dysfunction, including mitochondrial depolarization, swelling, impaired ATP synthesis, rupture of the outer mitochondrial membrane, and release of pro-apoptotic proteins [21,22,23]. While these mechanisms are well-documented, the precise molecular triggers of PTP induction in AD remain incompletely understood. Given ANT’s established role in PTP formation under conditions of calcium overload [23,31,32,33,35,47,52,53,54,55,56,57,58], its contribution to mitochondrial dysfunction in AD warrants further investigation. Importantly, the potential involvement of CRMP2 in modulating ANT-dependent PTP induction—and its implications for neuronal survival—had not been previously explored.

CRMP2 is a cytosolic phosphoprotein that regulates the localization and activity of various intracellular targets [60]. It is phosphorylated by kinases such as GSK-3β and Cdk5 [12,61,62,63], both of which are hyperactive in AD [7,8,9,10]. Elevated CRMP2 phosphorylation has been observed in postmortem AD brains [11,12,13,14,15] and in transgenic mouse models including APP/PS1 and Tg2576 [13,15,16,64], suggesting its involvement in early AD events [13]. In vitro, brief exposure of cultured cortical neurons to the toxic Aβ_25–35_ peptide fragment rapidly increases CRMP2 phosphorylation [65], highlighting its sensitivity to Aβ-mediated stress. Our previous work revealed a correlation between CRMP2 hyperphosphorylation, dissociation of phosphorylated CRMP2 from the ANT, and a consequent reduction in ANT transport activity [20]. Yet, other potential functional consequences of CRMP2 hyperphosphorylation have remained elusive. Our study addresses this gap by focusing on CRMP2’s emerging role in regulation of mitochondrial functions and its relevance to AD pathogenesis [18,20,51].

Indeed, CRMP2 has been shown to associate with mitochondria [17,18], with a subset localized to the intermembrane space [18], where it interacts with the ANT1 [17], a central regulator of mitochondrial bioenergetics [19]. Hyperphosphorylation of CRMP2 disrupts this interaction [17], and our findings confirm that this dissociation occurs in AD models [20]. However, the downstream consequences of CRMP2 disengagement from the ANT—particularly in the context of PTP induction—were previously unknown. We now show that preserving CRMP2-ANT interaction by preventing CRMP2 hyperphosphorylation can attenuate PTP induction, suggesting a protective role for CRMP2 in maintaining mitochondrial stability.

Our previous work demonstrated that ANT transport activity is compromised in synaptic mitochondria from APP-SAA KI mice, coinciding with CRMP2 hyperphosphorylation and its dissociation from the ANT [20]. Treatment with (S)-LCM restored CRMP2-ANT interaction and improved ADP/ATP transport across the inner mitochondrial membrane, supporting a model in which CRMP2 positively regulates ANT function. This was further validated using recombinant CRMP2 (rCRMP2) in ANT-reconstituted proteoliposomes, where rCRMP2 enhanced ADP/ATP exchange [20]. Interestingly, CRMP2 may not be the only protein capable of modulating ANT activity. Previous studies have shown that Bcl-2 can stimulate ANT function, while Bax exerts an inhibitory effect [66], suggesting a broader regulatory network involving the ANT and its interacting partners.

Although ANT’s role in ADP/ATP exchange is well established, its involvement in PTP formation has been debated [31,32,33,34,35]. Early studies proposed the ANT as a core component of the PTP [31,32,33,34,35], but the lack of definitive genetic evidence delayed consensus. Genetic ablation studies provided critical insights: Wallace’s group generated mice lacking ANT1 and ANT2 in liver mitochondria and found that PTP induction required significantly higher Ca^2+^ loads in ANT-deficient mitochondria compared to wild-type [67]. While the authors concluded that ANT is not essential for PTP formation, this interpretation is contentious. For instance, skeletal muscle mitochondria lacking ANT require an eightfold increase in Ca^2+^ to trigger PTP [68], which strongly suggests that ANT contributes to PTP induction. Rather than negating ANT’s role, these findings imply the existence of compensatory or parallel mechanisms. Indeed, recent studies implicating F_1_F_0_-ATP synthase in PTP formation [69,70] support a multifactorial model of PTP regulation.

The PTP triggered in isolated mitochondria (Figure 3 and Figure 4) may involve either the ANT, or ATP synthase, or both. In our experiments, the pronounced effects of BKA—a specific ANT inhibitor and negative modulator of the PTP [19,32,33,47]—strongly indicate that ANT plays a central role in PTP induction. Indeed, we previously found no evidence of CRMP2 binding to ATP synthase [20]. In contrast, unphosphorylated CRMP2 directly interacts with the ANT [20] (Supplementary Figure S2) and inhibits ANT-mediated channel activity (Figure 2). The ANT-associated ion currents identified in our study display strong voltage dependence (Figure 2). These currents remain open at low membrane potentials but progressively close as the potential becomes more positive. Our results indicate that rCRMP2 enhances voltage gating at lower voltages, thereby potentially limiting conditions that favor PTP opening.

Collectively, our data indicate that the CRMP2-dependent modulation of the PTP observed here is mediated primarily through ANT rather than ATP synthase. However, we cannot entirely exclude a contribution of the ATP synthase-associated PTP to mitochondrial dysfunction in AD. Previous studies have demonstrated that induction of the PTP in ANT-deficient cells requires substantially higher Ca^2+^ concentrations [67,68]. Consequently, it is conceivable that in AD higher Ca^2+^ may trigger both the ANT-associated and the ATP synthase-related PTP.

The increased susceptibility to PTP induction observed in AD models may have deleterious consequences for neuronal health. In our experiments, cortical neurons from APP-SAA KI (AD) cultured for 21 days (21 DIV) had lower viability compared to cultured cortical neurons from B6J hAbeta (Control). The neuronal death became apparent in both groups, but was significantly more pronounced in neurons derived from AD mice. Although the precise mechanisms driving this increased cell death remain to be elucidated, our data suggest a protective role for CRMP2 modulation. Pre-treatment with the small molecule (S)-LCM (10 µM, administered in the growth medium for 7 days prior to analysis) substantially improved neuronal survival in AD cultures. This neuroprotection correlated with inhibition of CRMP2 hyperphosphorylation at Thr 509/514 and Ser 522, reduced PTP induction, and enhanced mitochondrial resilience. Thus, our findings reveal a mechanistic connection between CRMP2 phosphorylation, PTP regulation, and neuronal survival in Alzheimer’s disease. These results highlight the critical need to identify molecular modulators—such as CRMP2—that could serve as therapeutic targets to maintain mitochondrial integrity and promote neuronal viability in AD.

## 5. Conclusions

We demonstrate that CRMP2 regulates mitochondrial PTP induction in APP-SAA knock-in mice, a mouse model of Alzheimer’s disease, through phosphorylation-dependent binding to the ANT. Unphosphorylated CRMP2 binds the ANT and suppresses PTP induction, whereas hyperphosphorylated CRMP2 dissociates from the ANT, increasing propensity to PTP induction and correlating with enhanced cell death in APP-SAA cortical neuronal cultures. (S)-lacosamide binds CRMP2, preventing GSK-3β- and Cdk5-mediated hyperphosphorylation at Thr509/514 and Ser522, thereby preserving CRMP2-ANT interaction and impeding PTP induction in APP-SAA mitochondria. These findings establish CRMP2 as a therapeutic target for preventing mitochondrial dysfunction and neurodegeneration in Alzheimer’s disease.

## Data Availability

Data are contained within the article or Supplementary Materials.

## Supplementary Materials

The following supporting information can be downloaded at: https://www.mdpi.com/article/10.3390/cells15020179/s1. Figure S1: CRMP2 phosphorylation is elevated in APP-SAA knock-in mice and reduced by (S)-LCM treatment, while total CRMP2 levels remain unchanged; Figure S2: CRMP2–ANT interaction in cortical synaptic mitochondria is disrupted in APP-SAA knock-in mice and restored by (S)-LCM treatment; Figure S3: (S)-lacosamide (10 μM) added directly to synaptic mitochondria isolated from cortices of APP-SAA KI mice (AD) failed to protect from Ca^2+^-induced mitochondrial swelling and depolarization, indicative of the permeability transition pore (PTP) induction; Figure S4: Ca^2+^ retention capacity of synaptic mitochondria isolated from cortices of 4-month old B6J hAbeta (Control, Ctrl) mice; Figure S5: (S)-lacosamide (10 μM) added directly to synaptic mitochondria isolated from cortices of APP-SAA KI mice (AD) failed to increase mitochondrial Ca^2+^ retention capacity.
