# Supplementary material for: Collapsin Response Mediator Protein 2 (CRMP2) Modulates Induction of the Mitochondrial Permeability Transition Pore in a Knock-In Mouse Model of Alzheimer’s Disease
Source: Cells. 2026 Jan 19;15(2):179. doi: 10.3390/cells15020179 (PMC12840006; doi:10.3390/cells15020179)
Supplement: Supplementary file 1 [file cells-15-00179-s001.zip › cells-4069781-supplementary.pdf]

SUPPLEMENTAL MATERIALS.

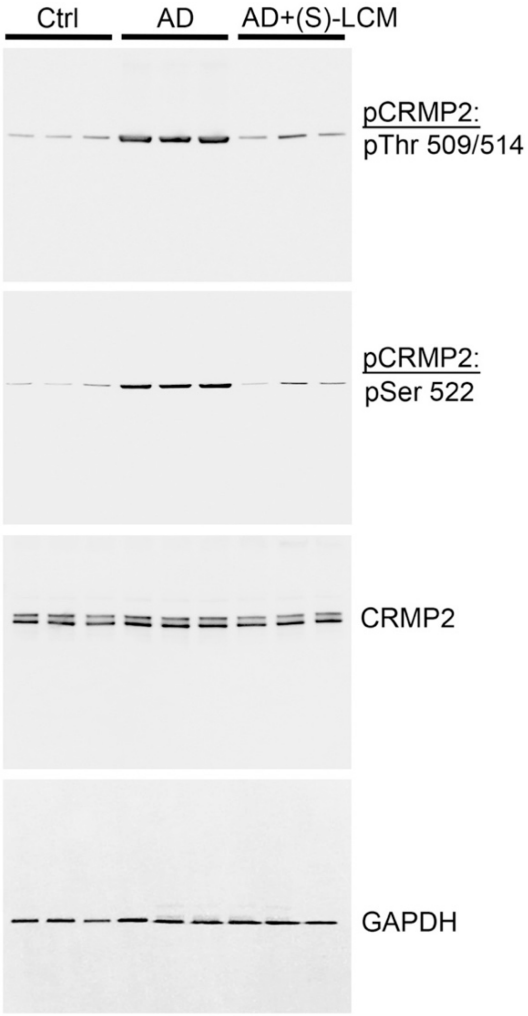

Supplemental Figure S1. **CRMP2 phosphorylation is elevated in APP-SAA knock-in mice and reduced by (S)-LCM treatment, while total CRMP2 levels remain unchanged.** Immunoblot analysis of cortical lysates from 4-month-old mice reveals increased phosphorylation of CRMP2 at Thr509/514 and Ser522 in APP-SAA knock-in mice (AD) compared to B6J hAbeta controls (Ctrl), while total CRMP2 expression remains unchanged across groups. Treatment with (S)-lacosamide ((S)-LCM; 10 mg/kg body weight, oral gavage for 7 days) significantly reduces CRMP2 phosphorylation in AD mice. Lanes 1–3 show samples from Ctrl mice; lanes 4–6, AD mice treated with vehicle (10 μL DMSO in 0.2 mL saline); lanes 7–9, AD mice treated with (S)-LCM. Phospho-specific antibodies targeting Thr509/514 and Ser522 were used to detect CRMP2 phosphorylation. Total CRMP2 was assessed using pan-CRMP2 antibody. Representative blots are shown from three independent biological replicates (N = 3). These results indicate that CRMP2 hyperphosphorylation is a feature of AD pathology and can be pharmacologically modulated by (S)-LCM.

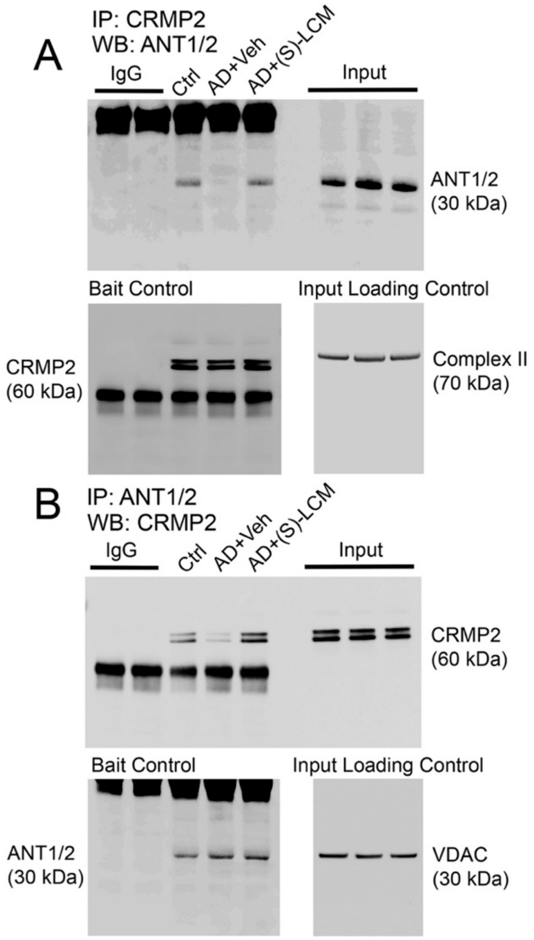

Supplemental Figure S2. **CRMP2–ANT interaction in cortical synaptic mitochondria is disrupted in APP-SAA knock-in mice and restored by (S)-LCM treatment.** CRMP2 associates with adenine nucleotide translocase (ANT) in synaptic mitochondria isolated from the cerebral cortex of 4-month-old B6J hAbeta mice (Ctrl). In age-matched APP-SAA knock-in mice (AD), this interaction is significantly reduced, indicating disease-associated dissociation. Treatment with (S)-lacosamide ((S)-LCM; 10 mg/kg body weight, administered via oral gavage for 7 consecutive days) prevents CRMP2 dissociation from ANT in AD mice. Panel A shows immunoprecipitation using anti-CRMP2 antibody, followed by immunoblotting with anti-ANT1/2 to detect co-precipitated ANT. Panel B presents the reciprocal experiment, with immunoprecipitation using anti-ANT1/2 antibody and detection of CRMP2. Vehicle-treated AD mice received 10 μL DMSO in 0.2 mL saline. Input lanes represent 5% of the total protein used in the pull-down assays. Bait protein controls and input loading controls are included where indicated. Synaptic mitochondria were isolated from both male and female mice. Representative immunoblots are shown from three independent biological replicates (N = 3). These data suggest that CRMP2–ANT interaction is sensitive to AD pathology and can be pharmacologically stabilized by (S)-LCM, highlighting a potential therapeutic mechanism targeting mitochondrial protein interactions.

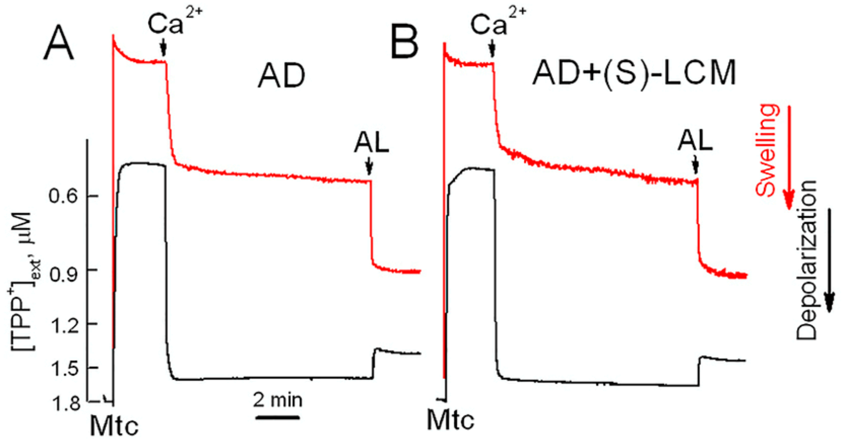

Supplemental Figure S3. **(S)-lacosamide (10 μM) added directly to synaptic mitochondria isolated from cortices of APP-SAA KI mice (AD) failed to protect from Ca^{2+}-induced mitochondrial swelling and depolarization, indicative of the permeability transition pore (PTP) induction.** Where indicated, 50 μM Ca^{2+} was added to induce the PTP. Alamethicin (AL) was used to induce maximal swelling.

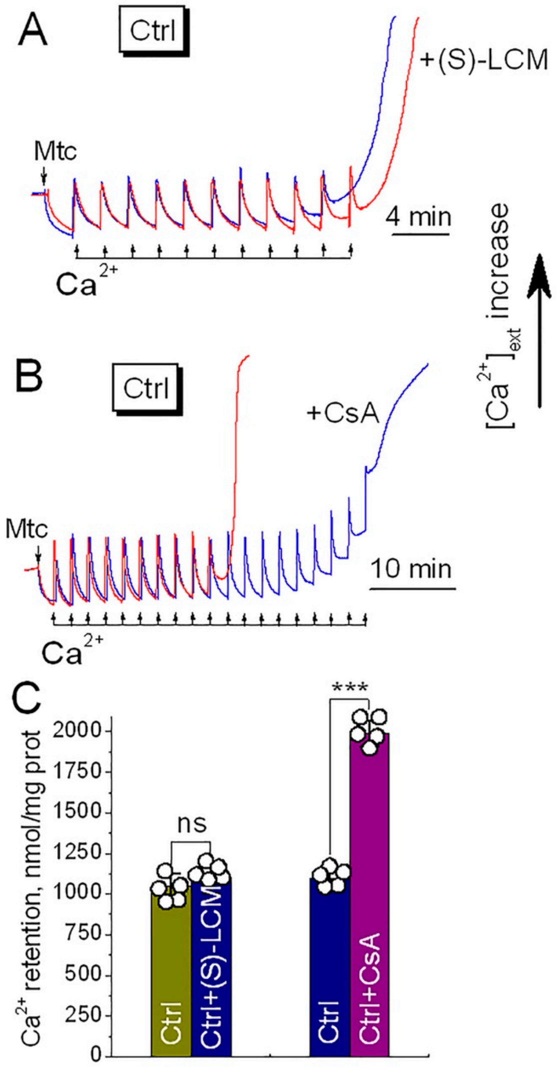

Supplemental Figure S4. **Ca^{2+} retention capacity of synaptic mitochondria isolated from cortices of 4-month old B6J hAbeta (Control, Ctrl) mice**. The Ca^{2+} retention capacity was evaluated as we described previously in the KCl-based incubation medium supplemented with 1 mM malate plus 3 mM pyruvate [38]. In **A**, synaptic mitochondria from **Ctrl** mice. Where indicated, mitochondria were isolated from **Control** mice pretreated with either a vehicle (Veh, 10 µl of DMSO in 0.2 ml saline) or 10 mg/kg (S)-LCM delivered by oral gavage for 7 days prior to mitochondrial isolation. In **B**, 1 µM cyclosporin A (CsA, positive control) was added to isolated mitochondria during the experiment. Ca^{2+} uptake and Ca^{2+} retention by mitochondria were monitored by following Ca^{2+} concentration in the medium ($[Ca^{2+}]_{ext}$) with a Ca^{2+}-selective electrode [38]. To assess mitochondrial Ca^{2+} retention capacity, multiple pulses of 10 µM Ca^{2+} were applied to mitochondria until a failure to take Ca^{2+} up and the release of accumulated Ca^{2+}. In **C**, statistical summary. Data are mean±SD, ***$p<0.001$; ns, not significant; N=5 separate experiments.

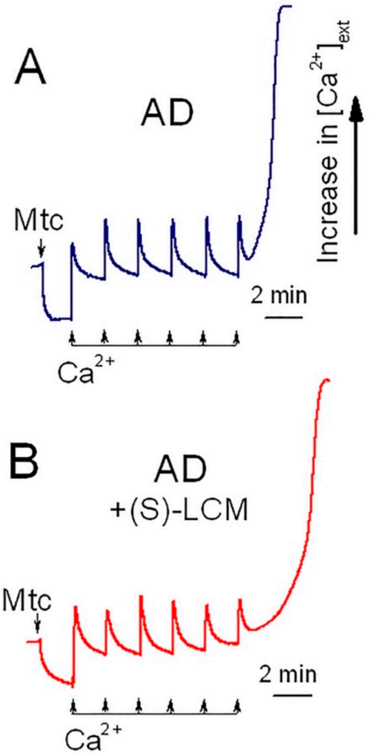

Supplemental Figure S5. **(S)-lacosamide (10 μM) added directly to synaptic mitochondria isolated from cortices of APP-SAA KI mice (AD) failed to increase mitochondrial Ca^{2+} retention capacity.** Where indicated, Ca^{2+} was added as multiple 10 μM $CaCl_2$ pulses.
